# Multiplatform Physiologic and Metabolic Phenotyping Reveals Microbial Toxicity

**DOI:** 10.1128/mSystems.00123-18

**Published:** 2018-11-06

**Authors:** Jingwei Cai, Robert G. Nichols, Imhoi Koo, Zachary A. Kalikow, Limin Zhang, Yuan Tian, Jingtao Zhang, Philip B. Smith, Andrew D. Patterson

**Affiliations:** a Center for Molecular Toxicology and Carcinogenesis, Department of Veterinary and Biomedical Sciences, The Pennsylvania State University, University Park, Pennsylvania, USA; b CAS Key Laboratory of Magnetic Resonance in Biological Systems, State Key Laboratory of Magnetic Resonance and Atomic and Molecular Physics, National Centre for Magnetic Resonance in Wuhan, Wuhan Institute of Physics and Mathematics, Chinese Academy of Sciences (CAS), Wuhan, China; c Metabolomics Facility, Huck Institutes of Life Sciences, Pennsylvania State University, University Park, Pennsylvania, USA; University of California, San Diego

**Keywords:** metabolomics, NMR, mass spectrometry, metabolism, toxicology, xenobiotic

## Abstract

The gut microbiota is susceptible to modulation by environmental stimuli and therefore can serve as a biological sensor. Recent evidence suggests that xenobiotics can disrupt the interaction between the microbiota and host. Here, we describe an approach that combines *in vitro* microbial incubation (isolated cecal contents from mice), flow cytometry, and mass spectrometry- and ^1^H nuclear magnetic resonance (NMR)-based metabolomics to evaluate xenobiotic-induced microbial toxicity. Tempol, a stabilized free radical scavenger known to remodel the microbial community structure and function *in vivo*, was studied to assess its direct effect on the gut microbiota. The microbiota was isolated from mouse cecum and was exposed to tempol for 4 h under strict anaerobic conditions. The flow cytometry data suggested that short-term tempol exposure to the microbiota is associated with disrupted membrane physiology as well as compromised metabolic activity. Mass spectrometry and NMR metabolomics revealed that tempol exposure significantly disrupted microbial metabolic activity, specifically indicated by changes in short-chain fatty acids, branched-chain amino acids, amino acids, nucleotides, glucose, and oligosaccharides. In addition, a mouse study with tempol (5 days gavage) showed similar microbial physiologic and metabolic changes, indicating that the *in vitro* approach reflected *in vivo* conditions. Our results, through evaluation of microbial viability, physiology, and metabolism and a comparison of *in vitro* and *in vivo* exposures with tempol, suggest that physiologic and metabolic phenotyping can provide unique insight into gut microbiota toxicity.

**IMPORTANCE** The gut microbiota is modulated physiologically, compositionally, and metabolically by xenobiotics, potentially causing metabolic consequences to the host. We recently reported that tempol, a stabilized free radical nitroxide, can exert beneficial effects on the host through modulation of the microbiome community structure and function. Here, we investigated a multiplatform phenotyping approach that combines high-throughput global metabolomics with flow cytometry to evaluate the direct effect of tempol on the microbiota. This approach may be useful in deciphering how other xenobiotics directly influence the microbiota.

## Abstract (precis)

The gut microbiota is modulated physiologically, compositionally, and metabolically by xenobiotics, potentially causing metabolic consequences to the host. We recently reported that tempol, a stabilized free radical nitroxide, can exert beneficial effects on the host through modulation of the microbiome community structure and function. Here, we investigated a multiplatform phenotyping approach that combines high-throughput global metabolomics with flow cytometry to evaluate the direct effect of tempol on the microbiota. This approach may be useful in deciphering how other xenobiotics directly influence the microbiota.

## INTRODUCTION

Microbes residing in the gastrointestinal tract can significantly influence host health by producing metabolites or molecules that function as available energy sources (e.g., short-chain fatty acid [SCFA]) (1, 2), metabolic signals (e.g., bile acid) (3, 4), and immune signals (e.g., lipopolysaccharides [LPS]) (5, 6). The gut microbiota is also involved in xenobiotic metabolism (e.g., drugs and environmental toxicants) through alteration of xenobiotic-metabolizing enzyme activity (7, 8), regulation of ligand-activated transcription factors, like the aryl hydrocarbon receptor (9, 10), or execution of biotransformation (11). Metagenomic and taxonomic tools have enabled the exploration of the diverse and complex microbial community structure. Additionally, metabolomics approaches, including mass spectrometry (MS)- and nuclear magnetic resonance (NMR)-based techniques, have provided valuable data to better understand connections between physiology and metabolism with respect to the host-microbiome interaction. However, measures of direct microbial toxicity caused by xenobiotic exposure have not been developed. Microbial toxicity assessment is important to understand the potential for drugs and other xenobiotics to influence the microbiota directly, especially xenobiotics that are not suspected of having antimicrobial activity.

**FIG 1:**
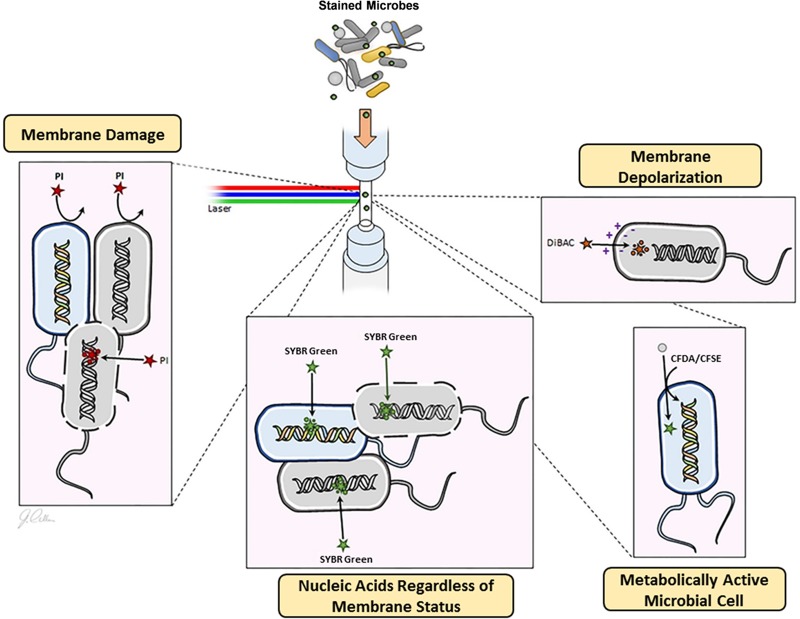
Flow cytometry provides a snapshot of the microbial physiological state. Four fluorescent dyes were used to evaluate different physiological parameters. Nucleic acid labeling with SYBR green indicates growth rate, bis-(1,3-dibutylbarbituric acid) trimethine oxonol (DiBAC) assesses membrane depolarization (moderate damage), propidium iodide (PI) determines loss of membrane integrity (severe damage), and carboxyfluorescein diacetate/carboxyfluorescein diacetate succinimidyl ester (CFDA/CFSE) measures metabolic activity.

Single-cell techniques, including flow cytometry, open the door for investigating the physiologic characteristics of microbial communities. Flow cytometric analysis has been employed to study the biochemical activity of microbial populations in environmental systems, including wastewater (12, 13) and aquatic ecosystems (14, 15), to characterize the physiology of the gut microbiota (16, 17) and to assess the microbial response to xenobiotics (18–20) and physical stress (21–23). Additionally, with the increasing popularity of anaerobic chambers, scientists have been able to appropriately culture and characterize a majority of microbes in complex environments, like the human gut (24). Utilizing the anaerobic chamber and flow cytometry, the physiologic and metabolic status of microbes can be characterized using different fluorescent dyes (Fig. 1).

Metabolomic approaches have been important for exploration of the metabolic effects of pharmaceuticals (25, 26), environmental contaminants (27, 28), and dietary factors (29, 30), as well as microbe-derived metabolites and microbiome-host cometabolites, to understand the metabolite chatter between the host and the gut microbiome (31–34). Mass spectrometry and NMR are widely used platforms for metabolomics, with each technique exhibiting its own merits and limitations (35, 36). Microbial metabolism determined by MS- and NMR-based metabolomics provides a functional readout of the microbial community, thus providing complementary insight into the characteristic changes in the metabolic activity, enzymatic pathways, and networks within the microbiota (37, 38).

**FIG 2:**
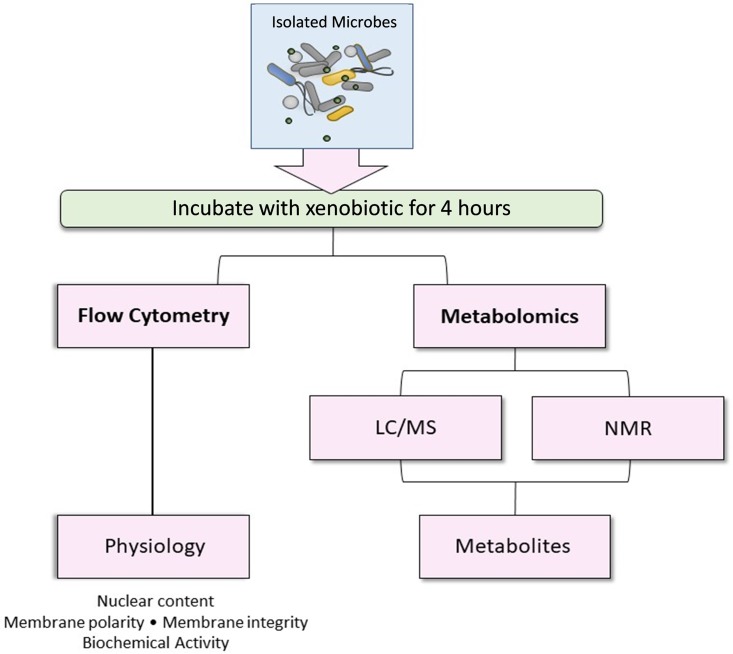
Experimental scheme of microbial toxicity assessment. Microbes isolated from mouse cecum were incubated with tempol for 4 h under strict anaerobic conditions (O_2_ < 20 ppm). Microbial toxicity was evaluated by characterizing microbial physiologic and metabolic status. Microbial physiologic status was evaluated by flow cytometry with different fluorescent dyes indicating membrane damage and biochemical activity. Microbial metabolism was assessed by global NMR- and LC-MS-based metabolomics.

Establishing microbial toxicity endpoints is key to understanding potential adverse interactions of xenobiotics with the microbiota. For example, tempol (4-hydroxy-2,2,6,6-tetramethylpiperidin-1-oxyl), an antioxidant reported to promote metabolic improvement (39) and attenuate metabolic dysregulation (4, 40), does so through alterations of the microbial community. However, the precise mechanism by which tempol interacts with the microbiome is not known. In the current study, gut microbiota toxicity was evaluated following short-term exposure to tempol (Fig. 2). ^1^H NMR and mass spectrometry-based global metabolomics and flow cytometry were performed to characterize the metabolic and physiological changes following tempol exposure. Combining measures of the microbial physiological state provided by flow cytometry and the metabolic status determined with metabolomics, this study revealed the direct effect of tempol on microbial physiology and metabolism. Importantly, this study supports the potential of physiological and metabolic phenotyping for the determination of microbial toxicity.

## RESULTS

### Short-term exposure of tempol *in vitro* directly impacts microbial physiology in a dose-dependent manner.

**FIG 3:**
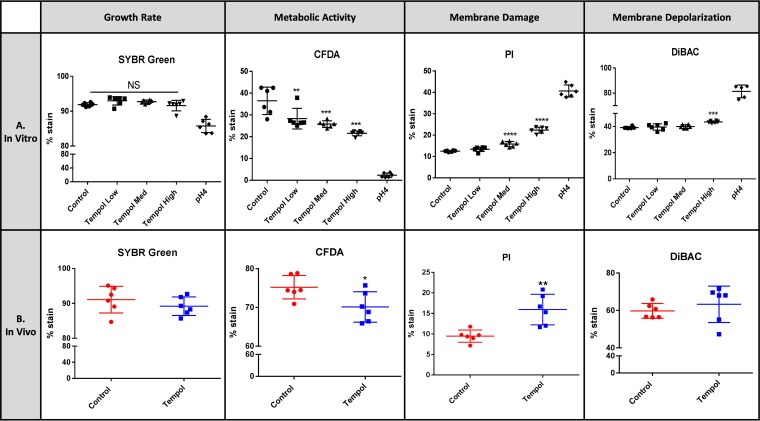
Tempol directly impacts microbial physiology. Microbial physiology was evaluated by flow cytometry after tempol exposure via 4-h short-term incubation of cecal microbiota with different doses of tempol (0.01, 0.1, and 1 mg/ml) *in vitro* (A) and 5-day gavage of tempol (100 mg/kg) to mice *in vivo* (B). **, *P* < 0.01; ***, *P* < 0.001; ****, *P* < 0.0001. One-way ANOVA with Tukey’s correction. The pH 4 group was introduced as a control. All data are presented as mean ± SD (*n* = 6 isolates per group). Med, medium.

Microbial physiology after short-term exposure of tempol *in vitro* was examined using a flow cytometry approach (Fig. 3A). Microbiota isolated directly from the mouse cecum was incubated in brain heart infusion (BHI) broth containing different doses of tempol (0.01 mg/ml, 0.1 mg/ml, and 1 mg/ml) under strict anaerobic conditions for 4 h at 37°C. A pH 4 group was introduced as a positive control by treating the microbiota with 12 M HCl. Acid treatment resulted in severe damage to microbial membranes, indicated by a significantly high percentage of propidium iodide-positive (PI^+^) cells (40.7% PI^+^ cells with pH 4 treatment versus 12.5% in control; *P < *0.001, Student’s *t* test) and bis-(1,3-dibutylbarbituric acid) trimethine oxonol-positive (DiBAC^+^) cells (81.3% DiBAC^+^ cells in pH 4 group versus 39.2% in control group; *P < *0.01, Student’s *t* test) (Fig. 3A). Additionally, the pH 4 group showed a decrease in SYBR green-stained cells (an average of 85.8% SYBR^+^ cells in pH 4 group compared to 91.9% in control group; *P < *0.05, Student’s *t* test) and drastically decreased metabolic activity, as revealed by a low percentage of CFDA^+^ cells (2.3% averaged CFDA^+^ cells in pH 4 group compared to 36.5% in control group; *P < *0.001, Student’s *t* test) (Fig. 3A). The pH 4 group with compromised physiologic and metabolic activity validated the feasibility of the flow cytometry method.

Flow cytometry analysis revealed a significant increase in DiBAC^+^ cells following high-dose tempol exposure (1 mg/ml) and a marked elevation of PI^+^ cells at medium and high doses of tempol (0.1 and 1 mg/ml, respectively) *in vitro*, indicating excessive membrane depolarization and a loss of membrane integrity of the microbiota (Fig. 3A). A significant decrease in CFDA^+^ cells was observed with all three doses of tempol compared to the control *in vitro*, suggesting that the metabolic activity of tempol-exposed microbiota was compromised (Fig. 3A). These data together demonstrated that tempol directly impacts membrane health and metabolic activity of the microbiota *in vitro*. Notably, the impact of tempol on microbial physiology *in vitro* is dose dependent, with a 100-fold dose range (0.01 to 1 mg/ml).

### Tempol directly alters microbial metabolism *in vitro*.

**FIG 4:**
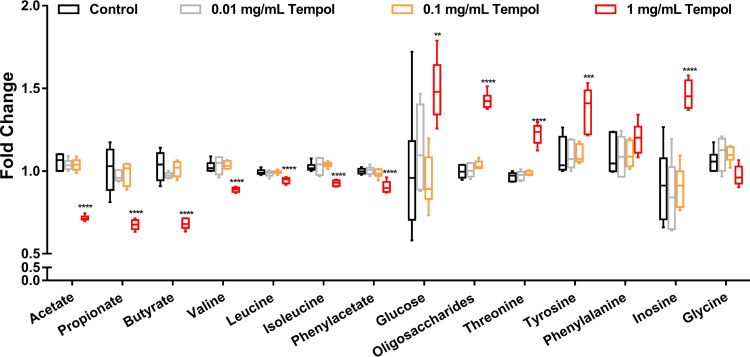
Tempol directly impacts microbial metabolic profiles characterized by ^1^H NMR. Relative concentrations of microbial metabolites after exposure to different doses of tempol *in vitro* measured via ^1^H NMR analysis. **, *P* < 0.01; ***, *P* < 0.001; ****, *P* < 0.0001 compared to control. One-way ANOVA with Tukey’s correction. All data are presented from minimum to maximum ranges with box and whisker plots (*n* = 6 isolates per group).

Metabolic profiling of tempol-exposed microbiota *in vitro* using ^1^H NMR metabolomics was performed. NMR analysis revealed a dose-dependent decrease in microbe-derived metabolites, including acetate, propionate, butyrate, valine, leucine, and isoleucine, suggesting the inhibition of microbial fermentation by direct tempol exposure (Fig. 4). In addition, energy metabolites and the fermentation substrates glucose and oligosaccharides were higher in concentration in microbiota exposed to high-dose tempol (Fig. 4). Moreover, a significant change in amino acid profiles characterized by a decrease of phenylacetate and an increase in aromatic amino acids, including tyrosine and phenylalanine, was identified (Fig. 4). The most versatile amino acid, threonine, which serves as a precursor for SCFAs synthesized by microbiota (41), was also significantly increased with tempol exposure (Fig. 4). Interestingly, the integration of physiological and metabolic biomarkers revealed a correlation of microbial physiology and metabolites following tempol exposure *in vitro* (see Fig. S1 in the supplemental material). Specifically, the inactivation physiological biomarkers (membrane damage indicators) PI and DiBAC are positively correlated with inactivation metabolic biomarkers (i.e., catabolism substrates), including glucose, oligosaccharides, and amino acids, while negatively correlated with activation metabolic biomarkers (i.e., catabolism products), including SCFAs and branched-chain amino acids (BCAAs) (Fig. S1). These data suggest that the compromised microbial metabolic activity is strongly correlated with the disrupted microbial membrane by direct tempol exposure.

10.1128/mSystems.00123-18.1 FIG S1 Correlations between microbial physiology and metabolism. Pearson correlation analysis was performed between physiological biomarkers and metabolic biomarkers characterized with flow cytometric and NMR analyses, respectively, and visualized using the heatmap.2 function from the gplots package in R. Red shades indicate a positive, while blue shades indicate a negative correlation. Download FIG S1, TIF file, 0.5 MB. Copyright © 2018 Cai et al. 2018 Cai et al. This content is distributed under the terms of the Creative Commons Attribution 4.0 International license.

### Comparison of physiologic and metabolic profiles of tempol-exposed microbiota *in vivo* and *in vitro*.

To further validate the viability of the described multiplatform approach *in vitro*, an *in vivo* exposure model was used by gavaging tempol to mice with a dose of 100 mg/kg of body weight (based on previous publications [39, 42]). The *in vivo* effect of tempol on microbial physiology and metabolism was analyzed following the same procedure performed for *in vitro* analysis. Consistent with the *in vitro* results, the microbial physiological status characterized by increased membrane damage (15.9% PI^+^ cells with tempol exposure versus 9.5% in control; *P < *0.01, Student’s *t* test) and decreased metabolic activity (70.1% CFDA^+^ cells with tempol exposure compared to 75.2% in control; *P < *0.05, Student’s *t* test) were observed *in vivo* with tempol treatment (Fig. 3B). Notably, the proportion of DiBAC^+^ cells, which showed a significant increase *in vitro*, remained unchanged *in vivo*.

**FIG 5:**
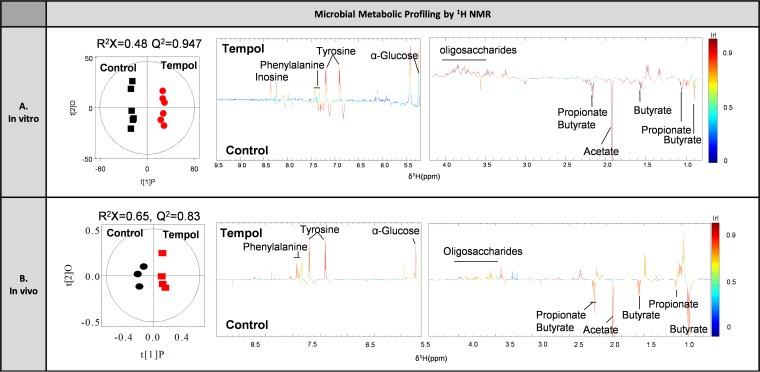
Similar microbial metabolic fingerprints between *in vivo* and *in vitro* exposures of tempol revealed by ^1^H NMR. OPLS-DA scores plots (left) indicating model quality and coefficient plots (right) displaying metabolite changes are generated using ^1^H NMR spectra from cecal microbiota after 4-h short-term incubation with tempol (1 mg/ml) *in vitro* (A) and cecal microbiota from mice gavaged with tempol (100 mg/kg) (B). The hotness of the color corresponds to the discrimination significance of the metabolite to the model separation. A positive peak (upward pointing) indicates that the metabolite is higher in concentration in the tempol treatment group, while a negative peak (downward pointing) indicates the metabolite is higher in concentration in the control group.

Microbial metabolic profiling after *in vivo* tempol exposure by ^1^H NMR revealed similar metabolic fingerprints with *in vitro* exposure, characterized by lower levels of SCFAs and BCAAs and higher levels of glucose and oligosaccharides (Fig. 5). The consistent results *in vitro* and *in vivo* suggested that the *in vitro* method could be a convenient alternative to study microbial toxicity.

### Tempol modulates microbial composition directly *in vitro*.

It is well established that microbial composition is intimately related to microbial functional roles and host metabolic outcomes. Having defined the physiological and metabolic changes in tempol-exposed microbiota *in vitro*, we then investigated the microbial community composition following tempol exposure *in vitro* by quantitative PCR (qPCR) analysis (Fig. S2). Total bacterial quantitation revealed a significant decrease in total bacterial population in medium and high doses of tempol *in vitro*, consistent with data reported from *in vivo* models (39). Consistent with previous *in vivo* studies of tempol (4, 39), significant decreases in Betaproteobacteria, Clostridium coccoides, Clostridium leptum subgroup, and Lactobacillus spp. were confirmed in the tempol-exposed *in vitro* model (Fig. S2). These data suggest that the observed microbial composition change in tempol-treated mice (39) is likely due, in part, to the direct impact of tempol on the microbiota.

10.1128/mSystems.00123-18.2 FIG S2 Tempol modulates microbial composition directly *in vitro*. Quantitative PCR analysis of 16S rRNA gene in the microbial isolates exposed to tempol *in vitro* with universal primers for absolute quantification of total bacteria with a standard curve (A) and targeted primers for relative quantitation of specific bacteria (B). *, *P* < 0.05; **, *P* < 0.01; ***, *P* < 0.001; ****, *P* < 0.0001 relative to control. One-way ANOVA with Tukey’s correction. All data are presented as mean ± SD (*n* = 6 isolates per group). Download FIG S2, TIF file, 0.4 MB. Copyright © 2018 Cai et al. 2018 Cai et al. This content is distributed under the terms of the Creative Commons Attribution 4.0 International license.

### Orbitrap LC-MS identified additional metabolic biomarkers within functional pathways and networks.

**FIG 6:**
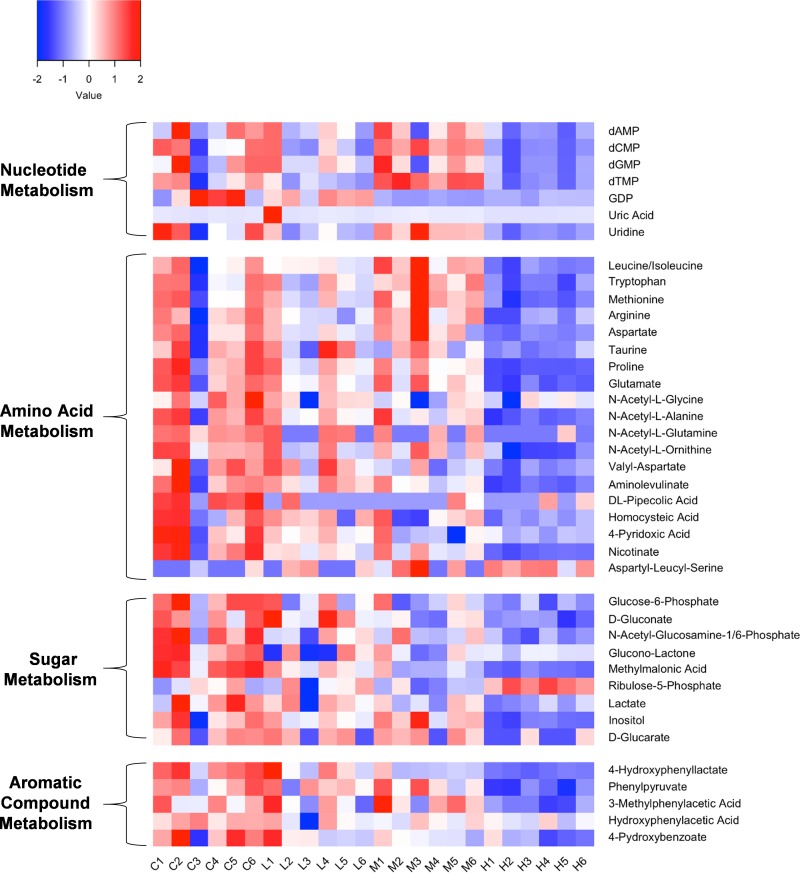
LC-MS revealed altered microbial metabolic profile in response to tempol. Z scores were created for the metabolomic data for each sample. Red shades represent metabolites that are increased or above the mean, and blue shades represent metabolites that are decreased or below the mean.

To provide an in-depth view of tempol-altered microbial metabolic features and pathways, liquid chromatography-mass spectrometry (LC-MS) was performed for additional metabolic biomarker identification and visualization of metabolic networks. Orbitrap LC-MS analysis identified over 40 significantly changed microbial metabolites in the tempol-treated group, providing additional metabolic biomarkers for microbial membrane damage and metabolism disruption following tempol exposure (Fig. 6). KEGG functional pathway analysis (Table S1) revealed a wide array of metabolic pathways altered by tempol exposure, especially nucleotide, amino acid, and sugar metabolism. Metabolite network visualization revealed a dose-dependent change in the tempol-treated microbiota (Fig. S3).

10.1128/mSystems.00123-18.3 FIG S3 Metabolic network changes of microbiome in response to tempol exposure. Network map was drawn with MetaMapp (for chemical and biochemical similarity) and Cytoscape (for visualization) using normalized LC-MS data (to internal standard). The blue and red of stand for significant down- and up-regulated compounds compared with control group, respectively, but green means no significant difference, which are calculated from *t* test. The size of node is dependent on the absolute value of the fold change. The orange peel (#FF9900) and egg blue (#00CCCC) colors stand for Tonimoto coefficient and KEGG reaction pairs obtained from MetaMapp package, respectively. Download FIG S3, TIF file, 2.1 MB. Copyright © 2018 Cai et al. 2018 Cai et al. This content is distributed under the terms of the Creative Commons Attribution 4.0 International license.

10.1128/mSystems.00123-18.4 TABLE S1 KEGG functional pathway analysis. Normalized LC-MS data (to internal standard) were mapped to the KEGG orthology database using MetaboAnalyst. The denominator and numerate are the number of compounds shown in each pathway and the number of compounds shown in the LC-MS data, respectively. The *P* value is estimated from global test based on hypergeometric test for the probability of having *n*-number of metabolites of a pathway in the input list, and the Holm method and FDR are applied for multiple comparisons. Here, the top-ranked pathways were selected for each comparison. Download Table S1, TIF file, 0.6 MB. Copyright © 2018 Cai et al. 2018 Cai et al. This content is distributed under the terms of the Creative Commons Attribution 4.0 International license.

Specifically, a significant downregulation of deoxyribose, ribose nucleotides, and derivatives, including dAMP, dCMP, dGMP, dTMP, GDP, uridine, and uric acid, were identified (Fig. 6). Nucleotides are used not only as backbone material for RNA and DNA synthesis but also as energy donors for many cellular functions, including amino acids, proteins, and cell membrane synthesis and transport (43). The downregulation of nucleotides with tempol exposure is in agreement with the altered aminoacyl-tRNA biosynthesis and amino acid, pyrimidine, and purine metabolism annotated by KEGG (Table S1). Moreover, a marked change in amino acid metabolism identified by KEGG pathway analysis was visualized using heatmaps (Fig. 6) and MetaMapp network visualizations (Fig. S3). High tempol exposure induced an overall decrease in essential amino acids (leucine, isoleucine, tryptophan, and methionine), nonessential amino acids (arginine, aspartate, taurine, proline, and glutamate), and amino acid derivatives (acetyl-glycine, acetyl-alanine, acetyl-glutamine, acetyl-ornithine, and valyl-aspartate) (Fig. 6). In addition, a significant decrease in glucose-6-phosphate was seen in microbiota exposed with all three different tempol doses, suggesting compromised glucose metabolism, as glucose-6-phosphate is the initiating molecule of glucose catabolism (44). Significantly decreased hydroxyphenylacetate, phenylpyruvate, and hydroxybenzoate revealed decreased microbial anaerobic metabolism of aromatic compounds (45, 46), consistent with the NMR results showing a decrease in phenylacetate and elevated aromatic substrates (Fig. 4).

## DISCUSSION

Evaluation of the potential toxicity of new drugs, xenobiotics, and/or toxicants to the gut microbiota may identify new mechanisms of toxicity and off-target effects and, ultimately, may help to improve risk assessment. This study took advantage of high-throughput metabolomics combined with flow cytometry to extensively investigate the physiologic and metabolic impact of tempol on the gut microbiota. The multiplatform phenotyping approach revealed a direct influence of short-term tempol exposure on microbial membrane health and metabolic activity, providing additional insight into the action of the xenobiotic tempol on the gut microbiota. Additionally, this study took advantage of the anaerobic chamber for rapid assessment of the effects of the xenobiotic tempol on the gut microbiome. While it is known that some gut microbial species are not culturable, this *in vitro* method provides a quantitative means of quickly testing microbial toxicity.

A human microbiome study demonstrated that membrane damage indicated by membrane depolarization and integrity loss was significantly increased with environmental hazards, like high heat and oxygen exposure (47). In the current study, we observed disruption of the microbial membrane as characterized by loss of polarity (DiBAC^+^) and integrity (PI^+^), suggesting that tempol directly targets the microbiota. Membrane physiology, including membrane potential, integrity, and energy metabolism, is critical for its overall function as well as to maintain passive permeability and active transport, extracellular/intracellular communication, and signal transduction (48). Thus, disrupted membrane physiology leads to profound and extensive physiologic and metabolic consequences, as demonstrated with tempol exposure.

In addition to physiologic biomarkers, metabolic biomarkers and the altered metabolic functional pathways with tempol exposure were identified using MS- and NMR-based metabolomics. The combination of MS- and NMR-based metabolomics permits a broader view of the metabolic alterations with improved metabolite identification and measurement confidence (36). Further, utilizing multiple platforms provides complementary metabolic views, thus providing greater confidence in data interpretation. NMR analysis identified significantly inhibited microbial fermentation and catabolic activity, as revealed by a decrease in SCFAs and an increase in fermentation substrates (glucose and oligosaccharides) and precursors (threonine), as well as a decrease in phenylacetate and an increase in aromatic amino acids, including tyrosine and phenylalanine. It has been reported that microbial enzymes and genes participate in aromatic amino acid catabolism, emphasizing the significant role of microbiota in phenylalanine and tyrosine degradation (49). Additionally, microbial enzymes are involved in the anaerobic oxidation of phenylalanine to generate phenylacetate (50). Therefore, the compromised microbial metabolism with low microbial enzyme activity after tempol exposure might explain the increase in aromatic amino acids phenylalanine and tyrosine and decrease in the degradation product phenylacetate in the metabolic profile.

Orbitrap LC-MS analysis identified over 40 significantly changed metabolites with tempol exposure in addition to NMR. Many of the metabolites are involved in critical metabolic pathways serving as critical metabolic biomarkers to indicate the profound systemic metabolic response of the gut microbiota to tempol exposure. For example, amino acids carry out important nutritional and physiologic roles in protein and coenzyme synthesis, cell signaling, and gene regulation (51). The microbiota is important for amino acid biosynthesis, catabolism, and utilization (41), and the gut microbiota contributes to the synthesis and consumption of different amino acids in response to environmental stress (51). For example, a microbial community, including Bifidobacterium adolescentis, Bacteroides thetaiotaomicron, Ruminococcus bromii, Eubacterium rectale, and Faecalibacterium prausnitzii, synthesizes higher levels of essential amino acids rather than nonessential amino acids (51). Given that tempol modulates microbial composition readily *in vivo* (4) and *in vitro* (Fig. S2), the alterations in amino acid profile are anticipated. In addition to amino acids, glucose-6-phosphate, which is an important initiator of glycolysis and the pentose phosphate pathway (44), was reduced, suggesting perturbed glucose metabolism after tempol exposure. Importantly, decreased glucose metabolism, as revealed by LC-MS is in agreement with the high level of sugar substrates, like glucose and oligosaccharides, is detected by NMR. Together, these findings suggest compromised microbial energy catabolism with tempol exposure.

The *in vitro* results are consistent with the previous and current findings *in vivo*. We have previously reported that tempol reduces microbial fermentation in mice (39). Consistent with the *in vivo* finding, a significant inhibition of microbial fermentation activity with short-term tempol exposure was observed *in vitro*, demonstrating that tempol modulation of the microbiota is likely through the direct action of tempol on the microbiota. Furthermore, tempol shifts host energy metabolism from energy storage to expenditure as an adaptive response to the reduced microbe-derived SCFAs *in vivo* (39). In the current study, a direct impact of tempol on microbial energy metabolism was identified *in vitro* as well.

Notably, the altered metabolic pathways are functionally interconnected. For example, nucleotides serve as both building blocks for nucleic acid synthesis and as energy donors for physiologic and metabolic functions. The altered nucleotide metabolism by tempol impacts microbial growth, which was revealed by total bacterial counts and disturbed energy metabolism. The compromised amino acid metabolism with tempol exposure could be either the result of the depleted energy supply due to disrupted nucleotide and carbohydrate metabolism or the cause of the compromised energy metabolism, as the nucleotide and carbohydrate metabolic pathways are heavily dependent on enzymes and cofactors, which are synthesized from amino acids.

The gut microbiota is susceptible to modulation from environmental toxicants and orally ingested xenobiotics, indicating that the gut microbiota is critical for understanding the complete mode of action and potential toxicity of xenobiotics and drugs. Recently, 40 representative human microbial strains were used to evaluate the impact of more than 1,000 nonantibiotic drugs on growth rate (52). Twenty-four percent of the drugs presented with antimicrobial or microbial modulatory effects, uncovering the potential risk of nonantibiotic drugs promoting antibiotic resistance. Further examination of these drugs could be completed with our approach combining both physiologic and metabolic measures and provide further mechanistic insight into their mode of action.

The gut microbiota can be associated with disease pathogenesis and is increasingly appreciated as a promising therapeutic target. Including the gut microbiota in toxicity assessment opens the door for better understanding the underlying mechanisms and potential off-target effects of xenobiotics. This work presented a novel multiplatform functional phenotyping approach that combines *in vitro* approaches, flow cytometry, and global metabolomics for integrated characterization of the physiological and metabolic phenotype of the microbiota in response to tempol exposure. This approach may further lead to the establishment of toxicity endpoints for the gut microbiota and ultimately provide a means to quantitatively and mechanistically assess the impact of other xenobiotics on the microbiota.

## MATERIALS AND METHODS

### Gut microbiota isolation and incubation conditions.

Six-week-old wild-type male C57BL/6J mice (The Jackson Laboratory, Bar Harbor, ME) were transferred into anaerobic chamber (Coy Laboratory Products, Inc., Grass Lake, MI) following CO_2_ asphyxiation. All the following procedures were performed under strict anaerobic conditions with an oxygen level below 20 ppm. The microbiota incubation and flow cytometry preparation procedures were derived and modified from a previously described protocol (47). Briefly, the cecal content was collected and diluted 1:10 (1 g in 10 ml) with brain heart infusion (BHI) broth (Sigma, St. Louis, MO). Each suspension was prepared in triplicate (one for the flow experiment and the other two replicates for MS- and NMR-based metabolomics analyses). The cecal content suspension was treated with tempol at final concentrations 0.01 mg/ml, 0.1 mg/ml, and 1 mg/ml, following a brief vortex and incubation at 37°C for 4 h in the dark. The negative-control group was treated with 12 M HCl to reach pH 4. After incubation, two sets of samples were stored at −80°C for future metabolomics analysis. The rest of the samples were centrifuged (700 × *g*, 4°C for 1 min). Six hundred microliters of the microbial supernatant was transferred to a new tube and then centrifuged (6,000 × *g*, 4°C for 3 min). The supernatant was discarded, and the microbial pellet was washed with prefiltered (0.2-µm-pore-size filter) reduced phosphate-buffered saline (PBS; 1× PBS solution containing 137 mM sodium chloride, 2.7 mM potassium chloride, 10 mM phosphate buffer, 1 mg/ml l-cysteine, and 1 µg/ml the oxygen indicator resazurin, an oxygen indicator), centrifuged (6,000 × *g*, 4°C for 2 min), and resuspended in 600 µl reduced PBS. The wash step was repeated two times until the microbial suspension was colorless. Then, the microbial cell suspension was diluted 120-fold with reduced PBS. Five hundred microliters of the diluted microbial suspension was transferred to a 1.5-ml tube to be stained for flow cytometry.

### Microbial physiology profiling with flow cytometry.

For a physiologic assessment of the microbiome after tempol exposure, we completed a flow cytometry analysis on incubated cecal contents. One critical physiologic parameter is nucleic acid content. Generally, cells with high nucleic acid content indicate more rapid transcriptional and metabolic activity and higher growth rate than do cells with low nucleic acid content (53, 54). Fluorescent dyes, like SYBR green, stain single- and double-stranded nucleic acids regardless of cell membrane status. The microbial membrane status is an excellent physiologic indicator of cellular damage, as loss of membrane integrity results in compromised selective permeability and functionality. Propidium iodide (PI) is a nucleic acid dye used to determine the viability of cells (55, 56), as it cannot penetrate an intact cell membrane due to its biochemical properties. Oxonol dyes, like bis-(1,3-dibutylbarbituric acid) trimethine oxonol (DiBAC), can be used to assess the loss of membrane polarity (57, 58), another indicator of cell damage. Normally, DiBAC is excluded from the cell, as both the dye and phospholipid membrane are negatively charged. Once the membrane is depolarized and loses membrane potential, DiBAC enters and binds to lipid-containing components. Another physiologic feature of bacteria is metabolic activity, which can be measured by fluorogenic esterase substrates, like carboxyfluorescein diacetate (CFDA) (12, 59, 60) and carboxyfluorescein diacetate succinimidyl ester (CFSE) (61). Fluorogenic esterase substrate is converted by intracellular esterase into fluorescein analogs, which are retained by cells. The strength of fluorescence corresponds to the enzymatic/metabolic activity.

Four distinct fluorescent dyes that stain cells based on nucleic acid content (SYBR green I, 1× final concentration, 15 min), membrane damage (PI, 40 µg/ml final concentration, 15 min; and DiBAC, 1 µg/ml final concentration, 10 min) and biochemical activity (CFDA, 10 µM final concentration, 30 min) were applied to the microbial suspension in the dark and under strict anaerobic conditions. All cytometric analyses were made using an Accuri C6 flow cytometer (Becton, Dickinson, Franklin Lakes, NJ) equipped with a solid-state 488- nm laser with standard filter setup. SYBR green I (488/520 nm), DiBAC (488/516 nm), and CFSE (488/517 nm) fluoresce in the green channel (FL1), and PI (488/620 nm) fluoresces in the yellow channel (FL2). Data were analyzed with the FlowJo software (version 10;). Cell growth and transcriptional activity were assessed by SYBR green, metabolic activity was indicated by CFDA, and PI and DiBAC were used as indicators of a compromised membrane and cell damage. Microbial suspension with a pH adjusted to 4 with 12 M HCl was used as a positive control for damaged microbial cells.

### ^1^H NMR metabolomics profiling.

The microbiota suspension saved after 4 h of incubation was used for ^1^H NMR spectroscopy. One milliliter of microbiota suspension was centrifuged at low speed (700 × *g*, 4°C for 1 min) to pellet down large particles. The maximum supernatant volume was transferred to a new tube and centrifuged at high speed (6,000 × *g*, 4°C for 3 min) to pellet down bacteria. The microbial pellet was washed two times with PBS. After the third wash, 1 ml of precooled methanol-H_2_O (2:1 [vol/vol]) and 1.0-mm-diameter zirconia/silica beads (BioSpec, Bartlesville, OK) were added to the microbial pellet, followed by homogenization (6,500 rpm, 1 cycle, 60 s) using the Precellys tissue homogenizer (Bertin Technologies, Rockville, MD). The homogenized sample was freeze-thawed three times with liquid nitrogen and a 37°C water bath, and then it was homogenized again and sonicated for 15 min at 250 W with a Branson 1510 ultrasonic cleaner (Branson Ultrasonics, Danbury, CT) to rupture microbial cell walls and release intracellular metabolites. The sample was centrifuged (11,180 × *g*, 4°C, and 10 min), and the supernatants were transferred to a new 2-ml tube. Another 1 ml methanol-H_2_O (2:1 [vol/vol]) was added to the pellets, and the extraction procedure was repeated. All supernatants were combined, dried down, and reconstituted in 600 μl of PBS (K_2_HPO_4_/NaH_2_PO_4_, 0.1 M [pH 7.4], containing 50% D_2_O and 0.005% [3-(trimethylsilyl)-2,2,3,3-tetradeuteropropionic acid] TSP-*d*4 as an internal standard). Following centrifugation (13,000 × *g*, 4°C, 10 min), 550 μl of each extract was transferred into a 5-mm NMR tube for analysis.

^1^H NMR spectra of extracted samples were acquired at 298 K on a Bruker NMR spectrometer (600 MHz for ^1^H) configured with a 5-mm inverse cryogenic probe, as previously described (9). In brief, a standard one-dimensional nuclear Overhauser enhancement spectroscopy (NOESY)–presaturation pulse sequence was employed, with irradiation at the water frequency during the recycle and mixing time delays to suppress the water signal. The 90° pulse length was adjusted to approximately 10 μs (9.6 dbW), and 64 transients were collected into 64 K data points for each spectrum, with a spectral width of 16 ppm. For the resonance assignment, a two-dimensional NMR spectroscopy was performed, including ^1^H–^1^H correlation spectroscopy (COSY), ^1^H–^1^H total correlation spectroscopy (TOCSY), ^1^H-^13^C heteronuclear single quantum correlation (HSQC), and ^1^H-^13^C heteronuclear multiple-bond correlation (HMBC) spectra.

^1^H NMR spectrum processing and multivariate data analysis were performed as previously described (36). Briefly, ^1^H NMR spectral quality was improved by phase, baseline correction, and calibration referenced to TSP-*d*4 (δ 0.0) using TopSpin 3.0 (Bruker BioSpin, Germany). The NMR spectra then were processed using the AMIX 3.9.14 software (Bruker BioSpin). The spectral region at δ0.5 to δ 9.0 was bucketed into 0.004-ppm bins. The residual water signal (region δ4.2 to 5.2) was discarded prior to normalization. The binned spectral data were normalized to the sum of the total intensity of the spectrum prior to the multivariate analysis. Multivariate data analysis was performed on the normalized binned NMR data with SIMCA 13 (Umetrics, Sweden). Principal-component analysis (PCA) was done first, with the score plots showing intergroup separation and the possible presence of outliers. Then, orthogonal projection to latent structures with discriminant analysis (OPLS-DA) was performed with a 7-fold cross-validation method using UV scaling. R^2^X and Q^2^ values generated from the model fitting represent the predictive power and validity of the models, respectively. The validation of the OPLS-DA model was further confirmed by cross-validated analysis of variance (CV-ANOVA) (implemented in SIMCA 13). To facilitate interpretation of the results, color-coded loading plots using Pearson linear correlation coefficients of variables from OPLS-DA loadings were generated by MATLAB (MathWorks, Inc., Natick, MA). The hotness of the color represents the significance of the metabolite contribution to intergroup separation, with a “hot/red” color being more significant than a “cold/blue” color. A cutoff value of |*r*| > 0.754 (*r* > 0.754 and *r* < −0.754) was used as significant based on the discrimination significance (*P ≤* 0.05).

### LC-MS metabolomics profiling.

Six hundred microliters of bacterial suspension after 4 h of incubation was centrifuged (700 × *g*, 4°C for 1 min), and supernatants were transferred to a new tube, centrifuged (6,000 × *g*, 4°C for 3 min), and washed 3 times with PBS. After the final centrifugation, 1 ml cold 50% aqueous methanol containing 1 µM chlorpropamide and 1.0-mm-diameter zirconia/silica beads (BioSpec, Bartlesville, OK) were added to the microbial pellet, followed with homogenization (6,500 rpm, 1 cycle, 60 s). The sample was freeze-thawed three times with liquid nitrogen to break apart the tough microbial cell wall. Then, the sample was centrifuged (maximum speed, 4°C, and 10 min), supernatants were transferred to a new Eppendorf tube, dried down, and resuspended in 200 µl of 3% aqueous methanol. After a final spin (maximum speed, 4°C, and 10 min), 150 µl of supernatants was transferred to an autosampler for LC-MS analysis.

Metabolomics profiling was performed with a Dionex Ultimate 3000 quaternary high-performance liquid chromatography (HPLC) pump, column compartment, and autosampler-coupled Exactive Plus Orbitrap mass spectrometer controlled by Xcalibur 2.2 software (Thermo Fisher Scientific, Waltham, MA). LC-MS was run with a modified ion pairing reversed-phase (RP) negative-ion electrospray ionization method (62). A total volume of 10 µl of sample is injected and separated on a Hydro-RP C_18_ column (100 by 2.1 mm, 2.5 µm particle size; Phenomenex, Torrance, CA) using a water/methanol gradient with tributylamine and acetic acid added to the aqueous mobile phase to enhance separation. The HPLC column is maintained at flow rate of 200 µl/min with the temperature of 30°C. The solvents and gradient are as follows: solvent A is 3% aqueous methanol with 10 mM tributylamine and 15 mM acetic acid, and solvent B is 100% methanol. The gradient is 0 min, 0% B; 5 min, 20% B; 7.5 min, 20% B; 13 min, 55% B; 15.5 min, 95% B; 18.5 min, 95% B; 19 min, 0% B; and 25 min, 0% B. The Exactive Plus is operated in negative-ion mode at maximum resolving power (140,000) and scans from *m/z* 72 to 1,000 for the first 90 s and then from *m/z* 85 to 1,000 for the remainder of the chromatographic run for the detection of small-molecule metabolites.

Orbitrap LC-MS data were analyzed with the open-source software pipeline MS-Dial (63). An in-house library generated from 288 pure metabolite standards was used for peak identification, with a strict accurate mass tolerance of 0.002 Da (5 ppm at mass of 400) and retention time (RT) tolerance of 0.5 min. Raw integrated data were normalized to chlorpropamide (*m/z* 275.0262; RT, 16.91 min). Filtering methods were applied to remove features with greater than 50% gap filling, and their peak areas were less than the background signal of blank injection. Chemical and biochemical similarities among identified compounds were calculated using MetaMapp (64). Biochemical mapping was calculated based on the KEGG reactant pair database, and chemical mapping was obtained from substructure comparison in the PubChem database using Tanimoto chemical similarity. For the visualization of biochemical and chemical mapping, Cytoscape was used. The *P* value from the statistical *t* test and fold change can show node color (related to up- and downregulation) and node size related to the absolute value of fold change. MetaboAnalyst (http://metaboanalyst.ca) (65) is used for metabolite set enrichment analysis, which identifies metabolite pathways using KEGG pathway information. For the metabolic profile visualized with a heatmap, Z-scores were created with the equation *z* = (*x* − *x*′)/[SD(*x*)], where *x* represents the individual metabolite level, *x*′ is the average value for the metabolite across all groups, and SD(*x*) is the standard deviation of the metabolite across all groups. All shown identified metabolites are significant at a *P* value of *<*0.05 from Student’s *t* test in high-tempol group relative to the control. Heatmaps were created with the heatmap.2 function from the gplots package in R.

### Quantitative PCR analysis. (i) Standard curve construction.

Escherichia coli (wild type [WT] strain MG1655) was cultured in Luria-Bertani medium at 37°C and 220 rpm in the incubator overnight. Spectrophotometer readings at an optical density at 600 nm (OD_600_) were obtained (Eon microplate spectrophotometer; Bio-tek) to estimate bacterial numbers. A series of diluted E. coli media (dilution degree is based on estimated bacterial numbers) were cultured on LB agar plates under the same conditions (24 h at 37°C). All plate cultures were analyzed in triplicate. The colony counts were averaged to determine the total bacterial number, represented as CFU. In parallel, DNA from the same E. coli culture was extracted using the E.Z.N.A. stool DNA kit (Omega Bio-tek). Quantitative PCR assays were carried out using 16S rRNA universal primers (8F, 5′-AGAGTTTGATCCTGGCTCA-3′; 338R, 5′-CTGCTGCCTCCCGTAGGAGT-3′) on serially diluted DNA with Fast SYBR green qPCR master matrix on an ABI Prism 7900HT Fast real-time PCR sequence detection system (Applied Biosystems, Foster City, CA). The reactions were analyzed according to the ΔΔ*C_T_* method. qPCR conditions were 95°C for 20 s, 95°C for 3 s, and 60°C for 30 s, for 45 cycles. A standard curve was constructed with the threshold cycle (*C_T_*) value versus the microbial number.

### (ii) Bacterial quantification.

Microbial DNA from cecal contents (50 mg) was extracted using the E.Z.N.A. stool DNA kit (Omega Bio-tek). DNA concentration was determined using a NanoDrop spectrophotometer and diluted in diethyl pyrocarbonate (DEPC) water at a concentration of 1 ng/µl. DNA was then subjected to quantitative PCR using Fast SYBR green with the indicated universal 16S rRNA primers, PCR conditions, and ΔΔ*C_T_* method described above. *C_T_* values were substituted into a standard curve. The final results were expressed as bacterial number per milligram of microbial pellet. For relative specific bacterial quantification, specific primers were utilized instead of the universal primer (Table S2).

10.1128/mSystems.00123-18.5 TABLE S2 Primer sequences of bacteria for qPCR, related to Fig. S2. Download Table S2, TIF file, 0.4 MB. Copyright © 2018 Cai et al. 2018 Cai et al. This content is distributed under the terms of the Creative Commons Attribution 4.0 International license.

### Statistical analysis.

Graphical illustrations and statistical analyses were performed using Prism version 6, Microsoft Excel (2016), and RStudio (1.1.419). All data values were expressed as the mean ± standard deviation (SD). Statistical significance was defined as a *P* value of *<*0.05. Pearson correlation analysis was used to investigate the relationships between stain intensity and metabolite levels across all three doses (low, medium, and high). Statistical significance was determined by transforming the Pearson *r* values into *t* values and then using *t* distributions to determine *P* values. The equation used to find the statistical significant cutoff was *r* = *t*/√(*t*^2^ + *n* − 2), where *r* is the correlation value and *n* is the number of subjects. In this experiment, *n* was equal to 24. The *t* value was found by using the Excel function tinv (0.05,22), where 0.05 represents a *P* value of 0.05 and 22 is the degrees of freedom for this experiment (*n* − 2). Results were shown using the heatmap.2 function from the gplots package in R.

### Data availability.

The mass spectrometry and NMR data have been deposited in the Metabolomics Workbench (http://www.metabolomicsworkbench.org/) under project ID PR000681. The data can be accessed directly via its project doi, https://doi.org/10.21228/M8NH4G.
