# Supplementary figures and images for: Multiplatform Physiologic and Metabolic Phenotyping Reveals Microbial Toxicity
Source: mSystems. 2018 Nov 6;3(6):e00123-18. doi: 10.1128/mSystems.00123-18 (PMC6222046; doi:10.1128/mSystems.00123-18)

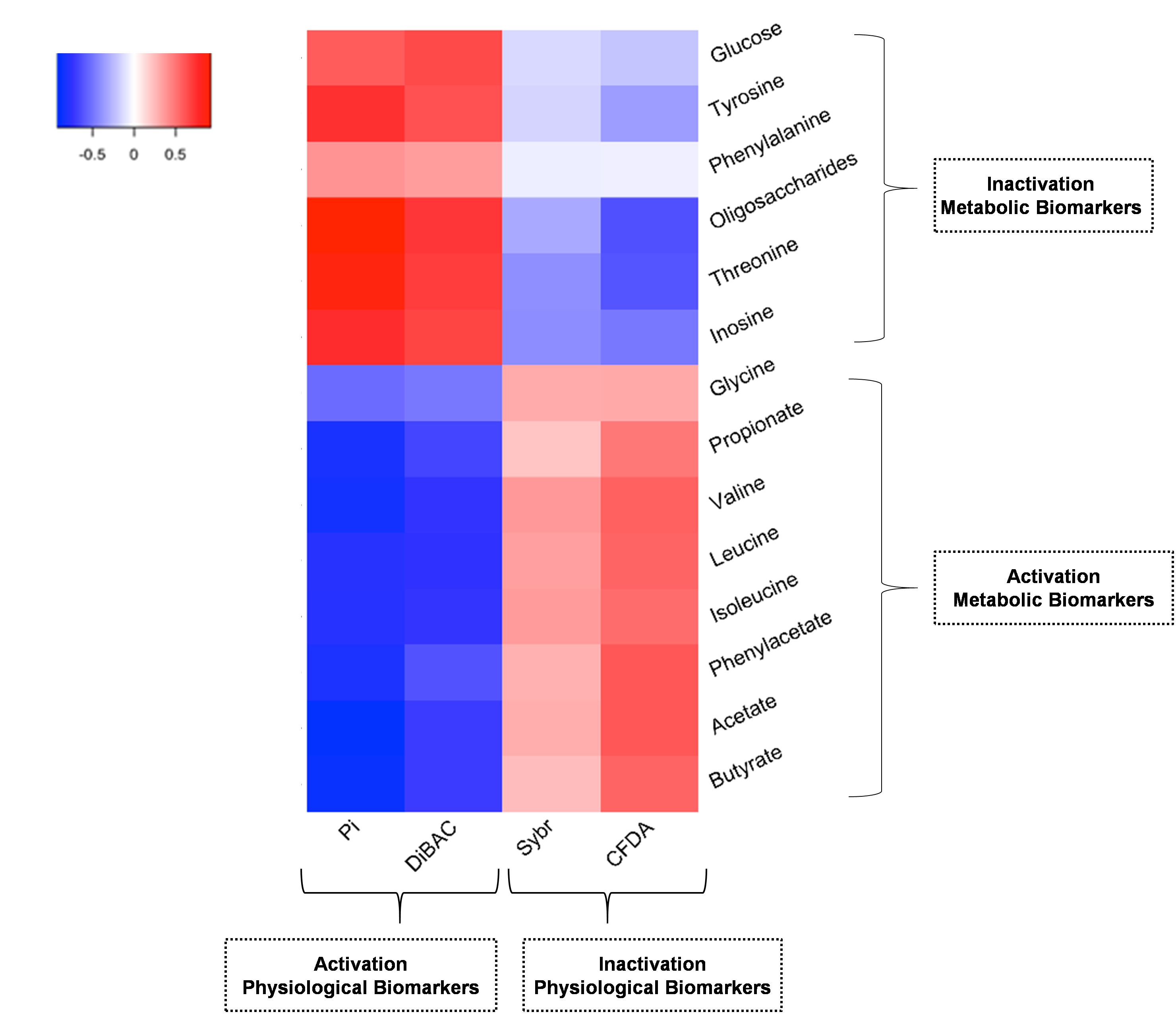

Supplement: FIG S1 [file sys006182284sf1.tif]

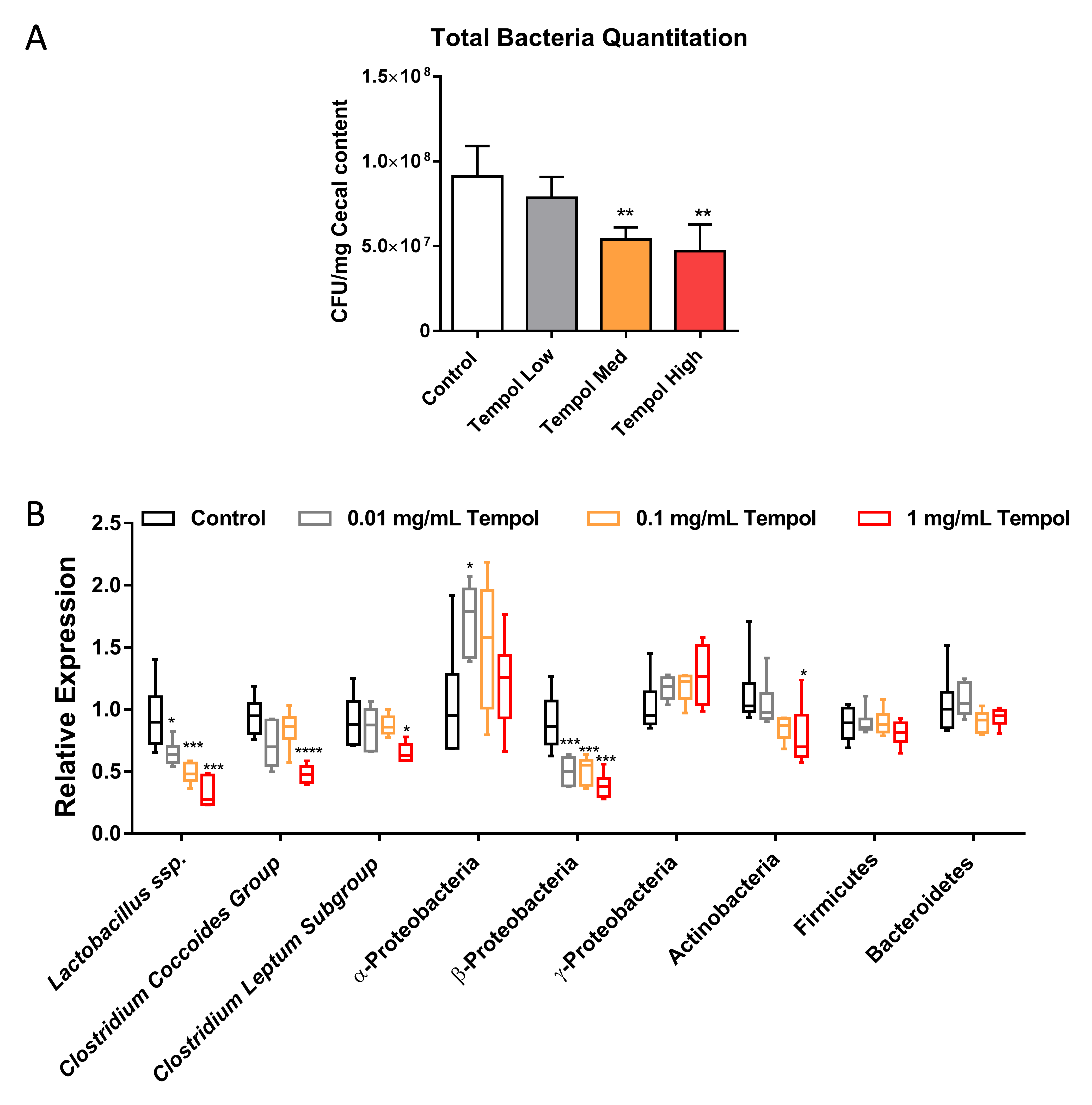

Supplement: FIG S2 [file sys006182284sf2.tif]

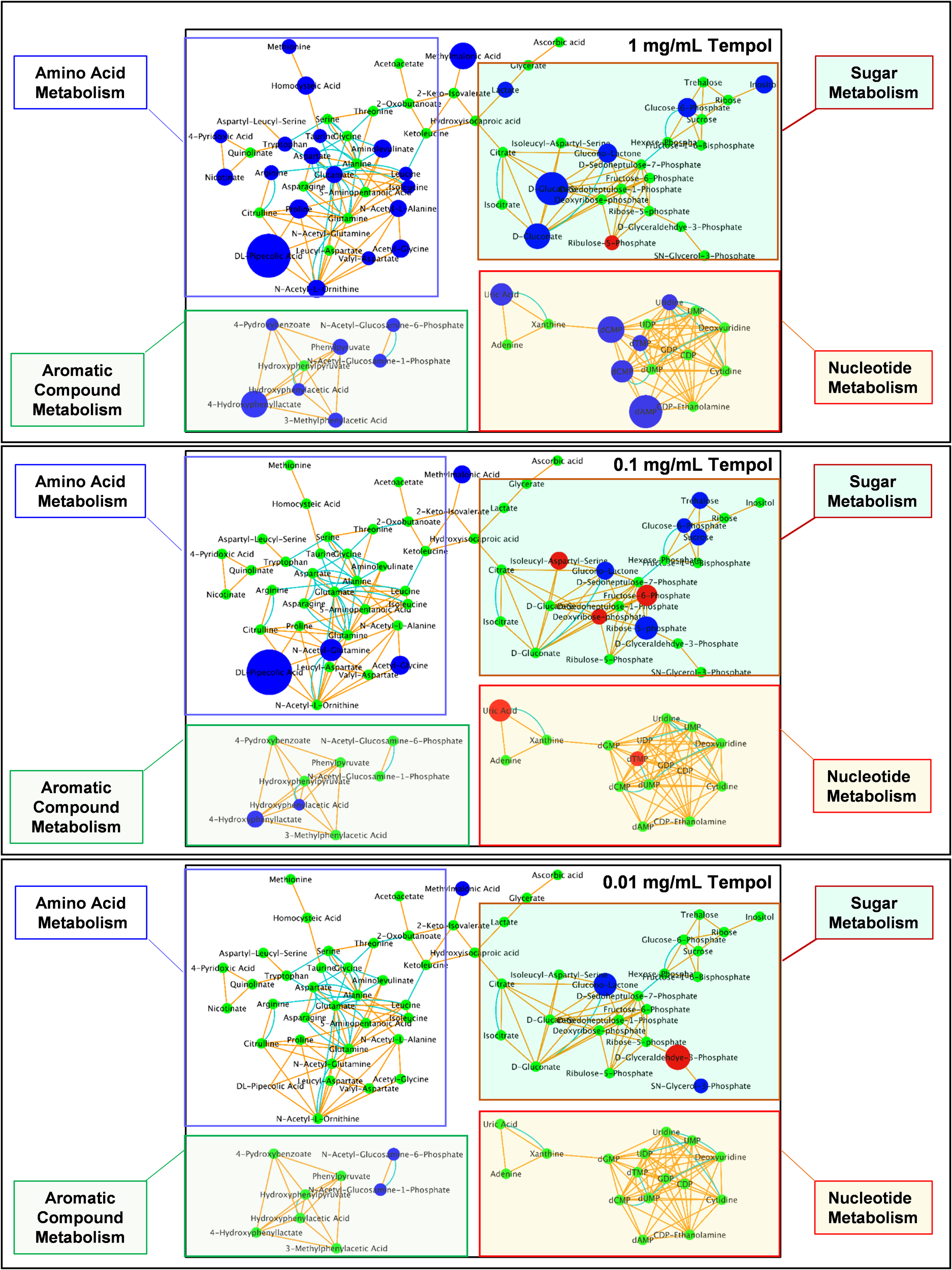

Supplement: FIG S3 [file sys006182284sf3.tif]

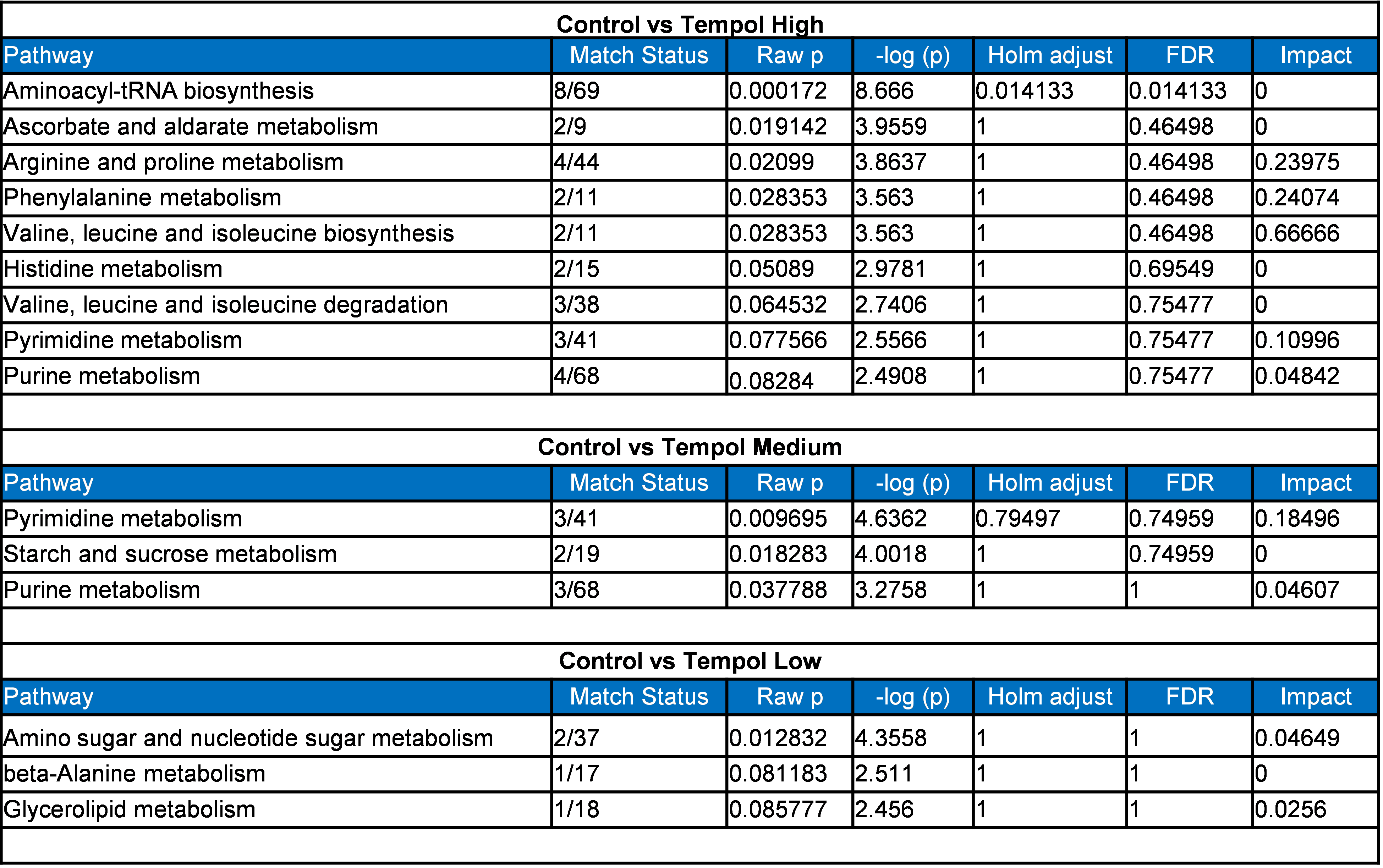

Supplement: TABLE S1 [file sys006182284st1.tif]

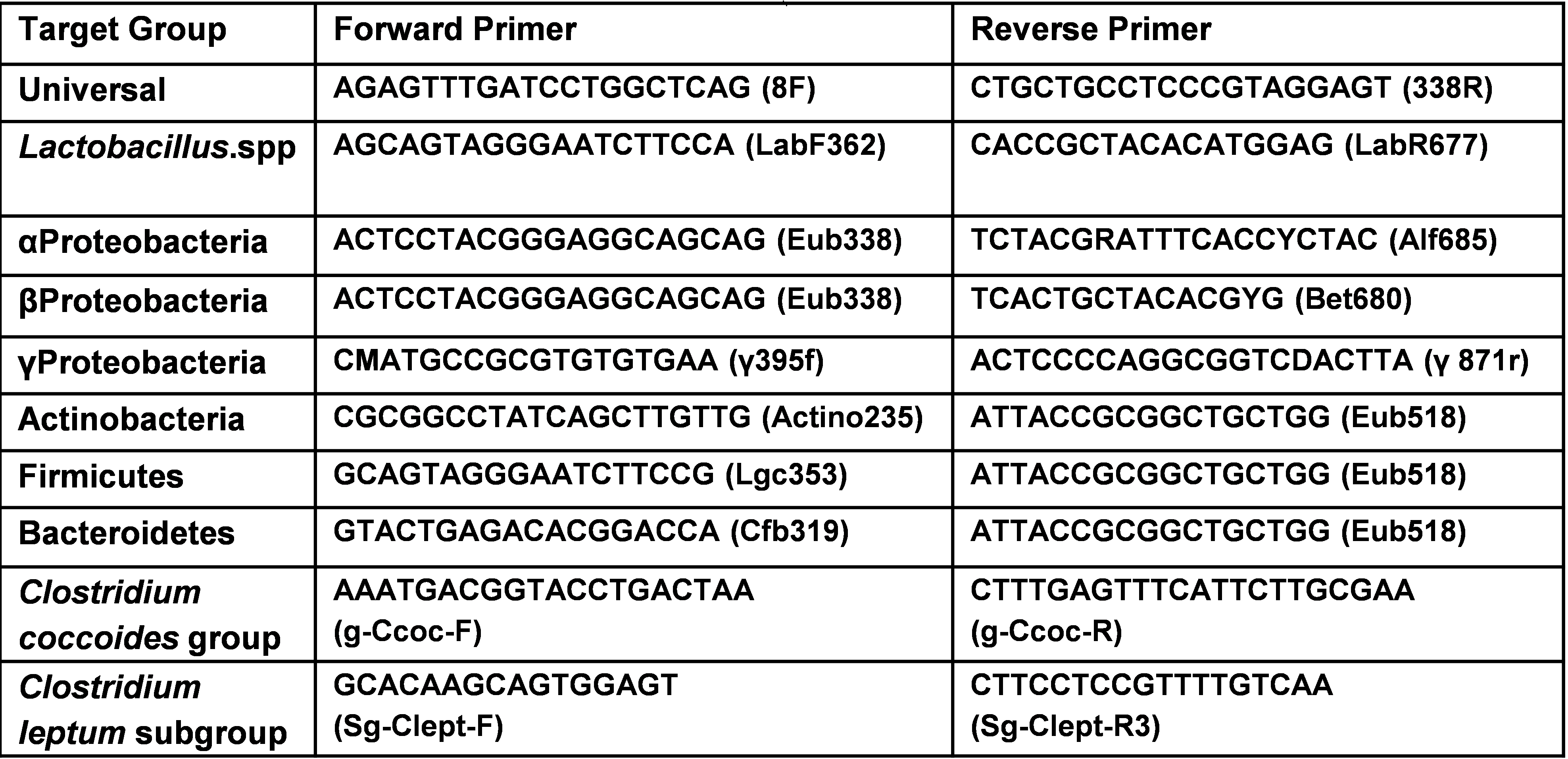

Supplement: TABLE S2 [file sys006182284st2.tif]
